# The Zinc Finger Protein Zbtb18 Represses Expression of Class I Phosphatidylinositol 3-Kinase Subunits and Inhibits Plasma Cell Differentiation

**DOI:** 10.4049/jimmunol.2000367

**Published:** 2021-02-19

**Authors:** Bin Xie, Tariq E. Khoyratty, Enas Abu-Shah, Pablo F. Cespedes, Andrew J. MacLean, Gabriela Pirgova, Zhiyuan Hu, Ahmed A. Ahmed, Michael L. Dustin, Irina A. Udalova, Tal I. Arnon

**Affiliations:** * Kennedy Institute of Rheumatology, University of Oxford, Oxford OX3 7FY, United Kingdom; and; † Ovarian Cancer Cell Laboratory, Weatherall Institute of Molecular Medicine, University of Oxford, Headington, Oxford OX3 9DS, United Kingdom

## Abstract

The PI3K pathway plays a key role in B cell activation and is important for the differentiation of Ab producing plasma cells (PCs). Although much is known about the molecular mechanisms that modulate PI3K signaling in B cells, the transcriptional regulation of PI3K expression is poorly understood. In this study, we identify the zinc finger protein Zbtb18 as a transcriptional repressor that directly binds enhancer/promoter regions of genes encoding class I PI3K regulatory subunits, subsequently limiting their expression, dampening PI3K signaling and suppressing PC responses. Following activation, dividing B cells progressively downregulated Zbtb18, allowing gradual amplification of PI3K signals and enhanced development of PCs. Human Zbtb18 displayed similar expression patterns and function in human B cells, acting to inhibit development of PCs. Furthermore, a number of Zbtb18 mutants identified in cancer patients showed loss of suppressor activity, which was also accompanied by impaired regulation of PI3K genes. Taken together, our study identifies Zbtb18 as a repressor of PC differentiation and reveals its previously unappreciated function as a transcription modulator of the PI3K signaling pathway.

## Key Points

- The Zinc finger protein, Zbtb18, is downregulated during PC differentiation.
- Enhanced expression of Zbtb18 leads to impaired PC development.
- Zbtb18 directly binds and inhibits expression of PI3K subunits.

## Introduction

Antibody responses are crucial for protection against invading pathogens and for providing long-term immunity in response to vaccination. However, the potential long-term persistence of plasma cells (PCs) also poses risks for developing pathogenic Ab responses and autoimmune diseases (1, 2). There is, therefore, considerable interest in defining specific factors and mechanisms that control the development of these cells (3–6). A major signaling pathway involved in this process is the PI3K cascade, which is rapidly induced in B cells in response to various types of activation (7–11). Previous studies have shown that loss of PI3K activity leads to impaired PC differentiation, whereas stronger PI3K signals are associated with increased PC responses (12–14). Progressive increase in chromatin accessibility of PI3K genes during the initial phase of activation has also been noted (3, 15). However, the mechanisms that facilitate changes in PI3K chromatin accessibility during activation and the relevance of this process to PC differentiation have not been explored. Moreover, despite being a key signaling pathway involved in multiple biological processes and human diseases, the transcription factors that control PI3K gene expression remain unknown.

In this study, we identify the zinc finger protein (ZFP) Zbtb18 as a transcriptional suppressor that binds promoter/enhancer elements of genes encoding class I PI3K regulatory subunits, consequently limiting their expression. We demonstrate that gradual downregulation of Zbtb18 during the early phase of B cell activation enhances PI3K expression and promotes PC differentiation, a function that we show to be conserved in both mouse and human B cells. To the best of our knowledge, this is the first transcription factor that has been shown to directly regulate expression of PI3K genes in immune cells. Because Zbtb18 is expressed in other cell types, the implication of these findings may go beyond a role for Zbtb18 in regulating B cell responses and could be important for understanding how this protein modulates PI3K signals in health and disease.

## Materials and Methods

### Human samples B cells isolation, in vitro culture, and RNA electroporation

Human blood samples from nonclinical and deidentified leukapheresis reduction cones were purchased from National Health Service at the University of Oxford and processed under ethics license record number 11/H0711/7. Human peripheral B cells were isolated by using the RosetteSep Human B Cell Enrichment Kit (catalog no. 15064; StemCell Technologies). For experiments assessing mRNA expression, cells were further sorted into three subsets: naive B cells (CD19^+^ IgD^+^ CD27^−^), memory B cells (CD19^+^ CD27^+^ CD38^−^), and plasmablasts/PCs (CD19^+^ CD27^+^ CD38^+^).

For electroporation of Zbtb18-IRES-GFP and GFP control mRNAs, total B cells were isolated as indicated above (>81% CD19^+^) and then rested overnight at a final concentration of 5 × 10^6^ cells per milliliter in RPMI 1640 medium supplemented with 10% heat-inactivated FBS, 10 mM HEPES, 2 mM L-glutamine, 1 mM sodium pyruvate, 0.1 mM MEM nonessential amino acids, and 50 U/ml of penicillin-streptomycin. Cells were then stimulated with a cytokine mixture, referred in this study as B cell stimulatory mixture, containing recombinant human IL-2 (50 U/ml; PeproTech), IL-4 (10 ng/ml; PeproTech), IL-21 (20 ng/ml; BioLegend), and BAFF (20 ng/ml; BioLegend). Forty-eight hours later, B cells were collected for transfection; 5 × 10^6^ cells were resuspended in OPTI-MEM (at 25 × 10^6^/ml) and transferred to a 2-mm cuvette (Bio-Rad Laboratories), and either electroporated with equimolar ratios of GFP (10 μg) or Zbtb18-IRES-GFP (30 μg) mRNA at 300 V and 1 ms using an ECM830 Square Wave Electroporator (BTX). Control GFP or Zbtb18-IRES-GFP mRNA were generated from pGEM vector using in vitro RNA Transcription Kits (mMESSAGE mMACHINE T7 ULTRA, AM1345; Thermo Fisher Scientific) and further purified by MegaClear Kit (AM1908; Thermo Fisher Scientific). Immediately after transfection, cells were resuspended in supplemented RPMI 1640 media, and 24 h later, GFP expression was assessed by flow cytometry and B cell stimulatory mixture was added to the cultures for additional 2 d to generate CD19^+^ CD27^+^ CD38^+^ plasmablasts/PCs.

### Mice and bone marrow chimeras

C57BL/6 (CD45.2^+^) or B6 Ly5.2 (CD45.1^+^) mice were purchased from Charles Rivers Laboratories. MD4 (16) (MGI:2384162) and Bcl-2tg (17) (MGI:3842923) mice have been described previously. Blimp1-Venus mice on a B6 background were obtained from M. Saitou (18).

To generate bone marrow (BM) chimeras, CD45.2^+^ B6 mice were lethally irradiated with 1100 rad split in two doses (4 h apart) and reconstituted with 1 × 10^6^ to 3 × 10^6^ BM cells from the indicated donors. Mice were analyzed 8–14 wk later. Mice were bred and maintained under specific pathogen–free conditions in accredited animal facilities at Kennedy Institute of Rheumatology, University of Oxford. Experiments were conducted in accordance with the U.K. Scientific Procedures Act (1986) under a project license authorized by the U.K. Home Office. Both males and females were used.

### Retroviral transductions

For overexpression of Zbtb18, we used the murine stem cell virus (MSCV2.2) retroviral vector, whereas transduction of short hairpin RNA (shRNA) was performed using LMP retroviral vector (MSCV-LTRmiR30-PIG, kindly provided by Prof. M. Huse from Sloan Kettering; Open Biosystems). Zbtb18 or shRNA sequences were cloned into the above vectors followed by an internal ribosome entry site (IRES) and an expression marker.

Splenocytes were transduced with the above vectors as previously described (19). Briefly, splenocytes were harvested in media containing 0.25 μg/ml anti-CD180 (RP-105, clone RP14; BD Biosciences). Twenty-four and forty-eight hours later, cells were spin infected for 1.5 h with 1 ml per well freshly made retroviral supernatant (1000G, 25°C, brake off). Cells were harvested either 1 d (for cells transduced to overexpress Zbtb18 using the MSCV2.2 retroviral vector) or 72 h (for shRNA transduction, using the MSCV-LTRmiR30-PIG vector) after second spin infection. When cells were transduced to overexpress Zbtb18/control and Pten/control shRNA, cells were harvested 72 h after the second spin infection.

BM transduction was performed as previously described (20). Briefly, CD45.1^+^ donors were injected i.v. with 3 mg of 5-fluorouracil (Sigma-Aldrich). Four days later, BM was harvested and cultured in the presence of rIL-3 (20 ng/ml), IL-6 (50 ng/ml), and mouse stem cell factor (100 ng/ml; PeproTech). BM cells were spin infected twice in 2 consecutive d with supernatants of the relevant retroviral vector. Between spin infections, cells were cultured with the above cytokine containing medium. One day after the last spin infection, cells (2.5 × 10^6^ to 5 × 10^6^ per mouse) were injected into lethally irradiated CD45.2^+^ C57BL/6 recipients.

For transduction of the mouse B cell lymphoma line WEHI-231 (CRL-1702; American Type Culture Collection), cells were seeded at 1 × 10^6^ cells per well in a 24-well plate followed by spin infection with a retroviral vector expressing the GFP reporter, as above. To generate a stable cell line, the transduced cells were sorted (as GFP^+^).

### Adoptive cell transfer and immunization

To test the effect of Zbtb18 overexpression on B cell responses in vivo, splenocytes from CD45.2^+^ donors were transduced ex vivo with control (empty) or Zbtb18-expressing MSCV2.2 retroviral vectors, carrying the reporter Thy1.1, or GFP. One day after the second spin infection, transduced splenocytes were cultured in the presence of the stroma cell line 40LB [a kind gift from K. Motoyama (21)] for 2 d. The 40LB cell line constitutively express BAFF and CD40L, providing activation and survival signals, which we find to significantly improve expression of retroviral constructs. The frequencies of Thy1 and GFP expression were analyzed by flow cytometry. Based on this information, the cells were mixed to achieve a 1:1 ratio of Thy1.1/GFP–expressing B220^+^ B cells. After mixing, cells were analyzed again by flow cytometry to confirm 1:1 ratio before being cotransferred i.v. (1× 10^6^ cells) into CD45.1^+^ MD4 hosts. The next day, recipients were i.p. immunized with ∼2 × 10^8^ SRBCs supplemented with LPS (50 μg/ml, *Escherichia coli* 0111:B4; Sigma-Aldrich). Twelve days later, spleens were collected and analyzed by flow cytometry.

### Flow cytometry, tissue digestion, and sort

For staining of human B cells, the following Abs were used: IgD-SA605 (IA6-2) CD19-PE (HIB19), CD138-PEcy7 (MI15), HLA-DR-PerCP (L243), CD38-A700 (HB-7), CD27-BV421 (M-T271), and propidium iodide solution (catalog no. 421301) from BioLegend. FcR-blocking regent (130-059-901) was from Miltenyi Biotec. To stain mouse cells, we used B220-PE (RA3-6B2), IgD-A700 (11-26c.2a), GL7-A647, GL7-PE, CD138-BV421 (281-2), B220-A700 (RA3-6B2), CD138-allophycocyanin (281-2), Ly5.2-PEcy7 (A20), IgM-PEcy7 (RMM-1) and Thy1.1-Pacific Blue (OX-7) from BioLegend. FAS-PEcy7 (Jo2) and IgG1-biotin (A85-1) were from BD Biosciences. IgG1-allophycocyanin (M1-14D12) was from eBioscience. p-Akt S473 was from Cell Signaling Technology (CST; D9E), and Alexa Fluor 647–conjugated F(ab′)_2_ donkey anti-rabbit IgG (H+L) was from Jackson ImmunoResearch (711-606-152). To stain Zbtb18-Myc, we used the anti-Myc Ab (Ab9132; Abcam) followed by a secondary anti-goat A647 (catalog no. A27018; Life Technologies). For intracellular staining, cells were of p-AKT and anti-Myc, cells were fixed, permeabilized, and stained following the protocol described previously (22). Analysis of flow cytometry data were conducted in FlowJo software (version 10.4.2).

To stain PCs from freshly harvested spleens, spleens were harvested and digested with RPMI 1640 containing 2% FBS, collagenase type 4 (0.5 mg/ml), and DNase I (10 μg/ml) for 30 min in a 37°C incubator while shaking. Immediately after digestion, enzymes were inactivated by adding 2 mM EDTA for 5 min and cells were isolated through a 70-μm cell strainer.

For all flow cytometry–based analysis, cells were stained with the fixable viability dye eFluor 780 (Life Technologies). When staining for PCs, cells were also blocked with anti-FcR for 20 min (CD16/CD32, clone 93; BioLegend) prior to additional stains. BD Fortessa X-20 was used for flow cytometry.

All sorts in this study were done using the Flow Cytometry Cell Sorter BD Aria III.

### PC differentiation and cell stimulation in vitro

To induce PC differentiation in vitro, cells were seeded at 1 × 10^6^ per ml in B cell medium containing either anti-CD40 (10 μg/ml, FGK4.5; BioXCell) and IL-4 (10 ng/ml; eBiosciences) or LPS (5 μg/ml). Cells were incubated for several days, as indicated. On day 2, the culture was replenished by adding half of the above dose of anti-CD40 and IL-4 or LPS. In some experiments, Foxo1 inhibitor (AS1842856; Calbiochem) was added to the medium.

To stimulate WEHI cells, anti-IgM (1 μg/ml, 115-006-075; Jackson ImmunoReasearch) and anti-CD40 (4.5 μg/ml, FGK4.5; BioXCell) were added to the medium for the indicated time points.

### Incubation with the 40LB cells line

In some cases, as indicated in the legends of the relevant assays, transduced splenocytes were incubated with the cell line 40LB [a kind gift from K. Motoyama (21)] to improve cell survival and to enhance stimulation. For this, ∼15 × 10^6^ 40LB cells were irradiated with 40 Gy. The irradiated cells were washed, seeded in a 10-cm dish, and added with ∼25 × 10^6^ transduced B cells in a total of 50 ml of 40LB media (RPMI 1640 supplemented with 10% FBS, 100 U/ml penicillin-streptomycin, 10 mM HEPES, 1 mM sodium pyruvate, 50 μM 2-ME) for the indicated time.

### B cell proliferation assay

For cell proliferation analysis, splenocytes cells were stained with the CellTrace Violet regent (5 μM per 10 × 10^6^ cells per ml; Invitrogen). Cells were labeled for 15 min 37°C in serum free medium followed by washing. Labeled cells were subsequently stimulated to induce PC differentiation in vitro for 3 d.

### RNA extraction and real-time PCR

RNA extraction was performed based on the RNEasy Mini Kit (Qiagen) followed by reverse transcription reaction using cDNA Reverse Transcription Kit (catalog no. 4368814; Applied Biosystems). Real-time PCR was performed using PrecisionFAST Blue LRSY (Primer Design) on a ViiA7 384-well real-time PCR detection system (Applied Biosystems). All gene expression levels were normalized to an internal housekeeping (hk) gene (RPL13A for human samples, HPRT and GAPDH for mouse samples) and calculated as 2^−(CThk−CTgene)^.

### Assay for transposase-accessible chromatin followed by deep sequencing and data processing

Assay for transposase-accessible chromatin followed by deep sequencing (ATAC-seq) was performed by an optimized Omini-ATAC protocol (23). Samples were quality checked for ATAC-seq–specific patterning on a bioanalyzer chip and were pooled at an equimolar ratio and sequenced on an HiSeq2500 using 75 bp paired-end chemistry in Welcome Centre for Human Genetics, Oxford. Raw sequencing reads were trimmed with Cutadapt prior to mapping to the mm10 version of the mouse genome using Bowtie2 with the following parameters–local–X 2000. PCR duplicates were removed with PicardTools; additionally, reads mapping to chrM, with MAPQ <10, and insert sizes >150 bp were removed prior to peak calling. Peaks were called with MACS2 with the following settings:–format BAMPE–nomodel–keep-dup all–mfold 5 50. Peaks found in at least two biological replicates were kept for further analysis, and merged peak set consisting of all discovered peaks was used for differential accessibility testing. Reads were counted over the merged peak set using BedTools and tested using DESeq2. Significantly differentially accessible peaks were deemed as having adjusted *p* values <0.05 (likelihood-ratio test), and adjusted *p* values <0.05, and fold change >1.5 (Wald test). Gene ontology was conducted using GREAT. DeepTools was used to generate normalized BigWigs from all biological replicates, which were merged for visualization in an integrative genomics viewer. De novo motif discovery was conducted on differentially accessible peaks using HOMER findMotifsGenome.pl with default settings and enrichment of discovered motifs at peaks was calculated with the annotatePeaks.pl command with the option: “-hist 5.” The data are deposited at the Gene Expression Omnibus database at https://www.ncbi.nlm.nih.gov/geo/query/acc.cgi?acc=GSE129657.

### Chromatin immunoprecipitation

Chromatin immunoprecipitation (Chip) assay was described previously (24). A total of 5 × 10^6^ to 10 × 10^6^ transduced flow cytometry–sorted cells (live B220^+^ CD138^−^ GFP^+^ or RFP^+^) were fixed, lysed, and immunoprecipitated by 2 μg of mouse IgG Ab (sc-2025; Santa Cruz Biotechnology), anti-Myc Ab (Clone 4A6, 05-724; MilliporeSigma), anti-H3K4me1 (ab8895; Abcam), anti-H3K4me3 (ab8580; Abcam), anti-H3K27me3 (ab6002; Abcam), or anti-H3K27Ac (ab4729; Abcam) conjugating with dynabeads (10004D; Life Technologies). Alternatively, for Myc Ab pull-down experiments, cells were resuspended directly in Chip lysis buffer (1% SDS, 10 mM EDTA, 50 mM Tris-HCL) after being fixed in 1% formaldehyde and rinsed in PBS, to reduce the loss or leak of Zbtb18 protein/DNA complex during the processing. After elution, the DNA was purified with QIAquick PCR Purification Kit (28106; Qiagen). Quantitative PCR (qPCR) reaction was performed with PrecisionFAST Blue LRSY.

### Western blot and immunoprecipitation

Western blot was performed as previously described (25). A total of 2 × 10^6^ flow cytometry–sorted transduced B cells (live B220^+^ CD138^−^ GFP^+^ or RFP^+^) were lysed in 80 μl of lysis buffer (no. 9803; CST) supplemented with proteinase inhibitor (P8340; Sigma-Aldrich). When stably transduced WEHI cells were used, ∼19 × 10^6^ total cells of each were lysed in RIPA buffer (89900; Sigma-Aldrich) at 30 mln cells per milliliter supplemented with proteinase inhibitor (P8340; Sigma-Aldrich). For immunoprecipitation of Myc-tagged proteins, 5 × 10^6^ sorted transduced B cells (GFP^+^) were lysed in 300 μl lysis buffer (no. 9803; CST) supplemented with proteinase inhibitor (P8340; Sigma-Aldrich). The total cell lysis was incubated with 4 μg of mouse IgG Ab (sc-2025; Santa Cruz Biotechnology) and 50 μl of A/G-Sepharose beads (GE17-0574-02; GE Healthcare) at 4°C for 3 h to reduce nonspecific binding. The beads were spun down and the lysate was subsequently incubated with 2 μg anti-Myc Ab (05-724; MilliporeSigma) at 4°C O/N, followed by addition of 20 μl of beads for 2 h at 4°C. The whole-cell lysis or immunoprecipitated fraction was separated by SDS-PAGE and transferred onto nitrocellulose membranes (162-0115; Bio-Rad Laboratories), immunoblotted with anti-p85 Ab (ABS234; MilliporeSigma), anti-p101 Ab (D32A5, no. 5569; CST), or anti-GAPDH (6C5, MAB374; MilliporeSigma), anti–p-AKT (Ser473; New England Biolabs), anti-AKT (pan) (C67E7; CST), anti-Myc (Ab9132; Abcam), or anti-GAPDH (6C5, MAB374; Merck Life Science). For secondary Abs anti-mouse 680RD (catalog no. 926-68070; LI-COR Biosciences), anti-rabbit 800CW (catalog no. 926-32211; LI-COR Biosciences), and anti-goat 800CW (925-32214; LI-COR Biosciences) were used. Membranes were developed and quantified by using the Odyssey Imaging Systems (LI-COR Biosciences).

### Immunofluorescent confocal microscopy

Transduced splenocytes (2 × 10^6^ to 5 × 10^6^) were sorted (live B220^+^ CD138^−^ GFP^+^) and fixed in 2% paraformaldehyde for 40 min on ice. After washing, cells were incubated in 0.5% Triton X-100 for 30 min at room temperature for permeabilization. Primary stain with goat anti-Myc Ab (ab9132; Abcam) and secondary stain with donkey anti-goat Alex-594 Ab (ab150132; Abcam) were performed at room temperature for 45 min to 1 h. After staining, cells were imaged using an Olympus FV1200 IX83 Confocal System, with the use of a ×40 objective.

### Analysis of online transcriptomic data

To identify the ZFPs regulated in both germinal center (GC) B cells and PC compared with naive and memory B cells, we performed differential expression analysis on downloaded expression matrices of GSE60927 (6), GDS1695 (26), and GSE23925 (27) using the R package limma (28). The list of ZFPs was downloaded from HGNC. We compared follicular B cells versus BM PC in GSE60927, naive versus PC and naive versus GC in GDS1695, and naive versus GC in GSE23925. The intersected group of differentially expressed ZFP genes (fold change ≥2 and false discovery rate [FDR] ≤0.05) across all the comparisons led to the identification of four genes that were downregulated and one gene that was upregulated in both PC and GC compared with naive and memory B cells.

### Statistical analysis

For most experiments, *p* values were calculated with unpaired two-tailed Student *t* test for two-group comparisons. For in vivo experiments (comparing PC, GC B cells, and follicular B cells within the same mouse) and Chip qPCR assay, *p* values were calculated with paired two-tailed Student *t* test. For human B cell differentiation assay, *p* values were calculated with ratio-paired two-tailed Student *t* test. Unless otherwise indicated, the data in figures are displayed as the mean ± SD. The *p* values <0.05 were considered significant, and the following values were delineated: *p < 0.05, **p < 0.01, ***p < 0.001, and ****p < 0.0001. Statistical analysis was performed with GraphPad Prism 7.

## Results

### Zbtb18 suppresses PC differentiation

To identify candidate factors involved in regulation of PC development, we used existing published RNA-sequencing datasets (6, 26, 27) to search for genes that are differentially up- or downregulated in PCs and GC B cells compared with naive and memory B cells (≥2-fold change; *p* ≤ 0.05). We hypothesized that genes that are differentially expressed in both subsets are more likely to reflect factors that affect the early phase of PC differentiation, prior to entry to the GC reaction. We focused our analysis on zinc finger molecules because of their known function as transcriptional regulators of lymphocyte differentiation and maturation (29, 30). Among 932 ZFPs compared, we identified four downregulated (Klf2, Zbtb18, Morc3, and Trps1) and one upregulated (Tcf19) gene fitting the above criteria (Supplemental Fig. 1A, 1B). Because a role for Klf2 in regulation of B cell differentiation has been previously described (31–34), we focused our attention on Zbtb18, which was the second most significantly differentially expressed gene in our analysis.

**FIGURE 1.**
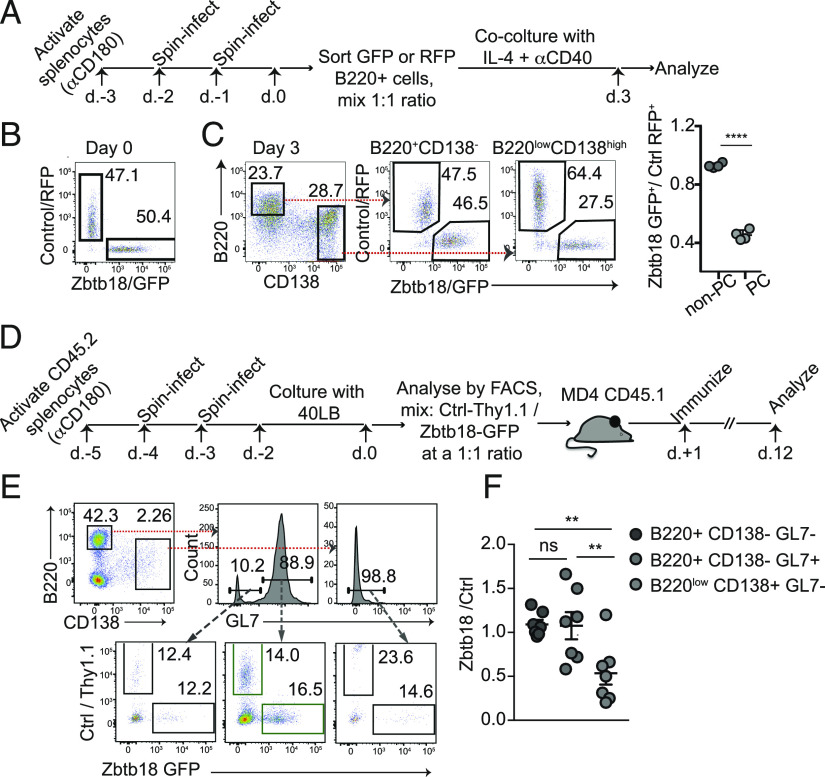
Zbtb18 suppresses PCs responses. (**A**–**C**) Splenocytes were transduced with Zbtb18/GFP or control/RFP retroviral vectors. B220^+^ RFP^+^ and GFP^+^ live cells were sorted by flow cytometry, mixed in a 1:1 ratio (day 0), and incubated with anti-CD40 and IL-4 for 3 d. A schematic illustration of the experiment is shown in (A). An example for flow cytometry gating scheme at day 0 (B) and day 3 (C) are shown. Red arrows show gating strategy. The ratio between Zbtb18/GFP^+^ and Ctrl/RFP^+^ cells in the non-PCs (B220^high^ CD138^−^) and PCs (B220^low^ CD138^high^) compartments is shown (C, right). (A)–(C) show the result of one representative experiment out of at least three independent experiments performed. Each circle represents one technical replicate. (**D**–**F**) CD45.2^+^ splenocytes were transduced with control/Thy1.1 or with Zbtb18/GFP and then cultured in the presence of 40LB stroma cells for 2 d. Transduced cells were analyzed by flow cytometry and cotransferred in a 1:1 ration into CD45.1^+^ MD4 mice. The next day, mice were immunized with SRBCs, and 12 d later, their spleens were analyzed by flow cytometry. A schematic illustration of the experiment is shown in (D). The gating scheme is shown (plots are pregated on live CD45.1^neg^ cells) (E). The ratios between CD45.2^+^ Zbtb18/GFP– and CD45.2^+^ control/Thy1.1–expressing cells were determined in the indicated populations (F). (D)–(F) show pooled data from two independent experiments performed. Each circle represents data from one mouse. **p < 0.01, ****p < 0.0001.

A role for Zbtb18 in regulating immune responses has never been reported. To first test whether it may be involved in PC differentiation, we enforced constitutive expression of Zbtb18 in B cells and determined its impact on the development of PCs. For this, splenocytes were retrovirally transduced with Zbtb18 or control MSCV vectors containing bicistronic GPF or RFP reporters, respectively. Overexpression of Zbtb18 in transduced B cells was confirmed by qPCR, indicating ∼2- to 3-fold increase compared with naive freshly isolated B cells or control-transduced cells (Supplemental Fig. 1C). Transduction of splenocytes with a Zbtb18-Myc–tagged construct further indicated that expression of the retrovirally transduced Zbtb18 protein tightly corelates with that of the reporter gene (Supplemental Fig. 1D). It should be noted that transduction of primary B cells involves initial stimulation of the cells with anti-CD180 Ab. However, this activation alone is not enough to drive PC responses. To test whether Zbtb18 overexpression affects PC differentiation, control/RFP– and Zbtb18/GFP–transduced B cells were sorted, mixed in a 1:1 ratio, and coincubated with IL-4 and anti-CD40 (see schematic illustration of experimental set up in Fig. 1A). Three days later, we determined the relative representation of Zbtb18/GFP– and control/RFP–transduced cells in the PC pool (B220^low^CD138^+^) and in the B220^+^CD138^−^ B cell subset (referred to as “non-PCs”). We found that whereas the ratio between Zbtb18- and control-transduced B cells in the non-PC subset remained constant and close to the initial 1:1 input, in the PC subset, Zbtb18-transduced cells were significantly outcompeted by control-transduced cells, and their relative representation dropped by more than 50% (Fig. 1B, 1C). A similar reduction in PC frequencies in Zbtb18-overexpressing cells was observed following stimulation with LPS (Supplemental Fig. 2A) or when cells were transduced with an alternative isoform of Zbtb18 (isoform 2) (Supplemental Fig. 2B). In this isoform, alternative splicing leads to the expression of a shorter protein lacking the first 9 aa at the N terminus of the protein. Notably, however, although Zbtb18 overexpression greatly reduced PC formation, it did not completely block their development or impact their ability to class switch (Supplemental Fig. 2C).

To further assess the relevance of these findings in the more complex setting of immune responses, we tested the effect of Zbtb18 overexpression in vivo. Wild-type (WT) CD45.2^+^ splenocytes transduced with Zbtb18/GFP or control/Thy1.1 vector. To increase cell survival and stimulation, transduced cells were incubated with the stroma cell line 40LB 2 d prior to harvesting (schematic illustration in Fig. 1D). Although this treatment does not lead to in vitro development of PCs, it further adds to the activation status of the transduced cells, possibly enhancing their ability to participate in the immune response in vivo. After 2 d, the cells were cotransferred into CD45.1^+^ MD4-recipient mice. We used MD4 animals that express a transgenic BCR with high affinity to lysozyme to reduce competition between transferred and endogenous B cells, thus allowing us to track the response of adoptively transferred polyclonal B cells. Twelve days postimmunization, we found that whereas the ratio between Zbtb18- and control-transduced cells was consistent and close to 1:1 within the non-PC subsets (including B220^+^ CD138^−^ GL7^−^ and GL7^+^ cells), in the PC pool (B220^+^ CD138^+^ GL7^−^), Zbtb18-overexpressing cells were outcompeted and their frequencies were reduced by ∼50% compared with control-transduced B cells (Fig. 1E, 1F). Together, these findings reveal a significant and intrinsic role for Zbtb18 in suppressing PC responses.

### Downregulation of Zbtb18 correlates with cell division and PC differentiation

**FIGURE 2.**
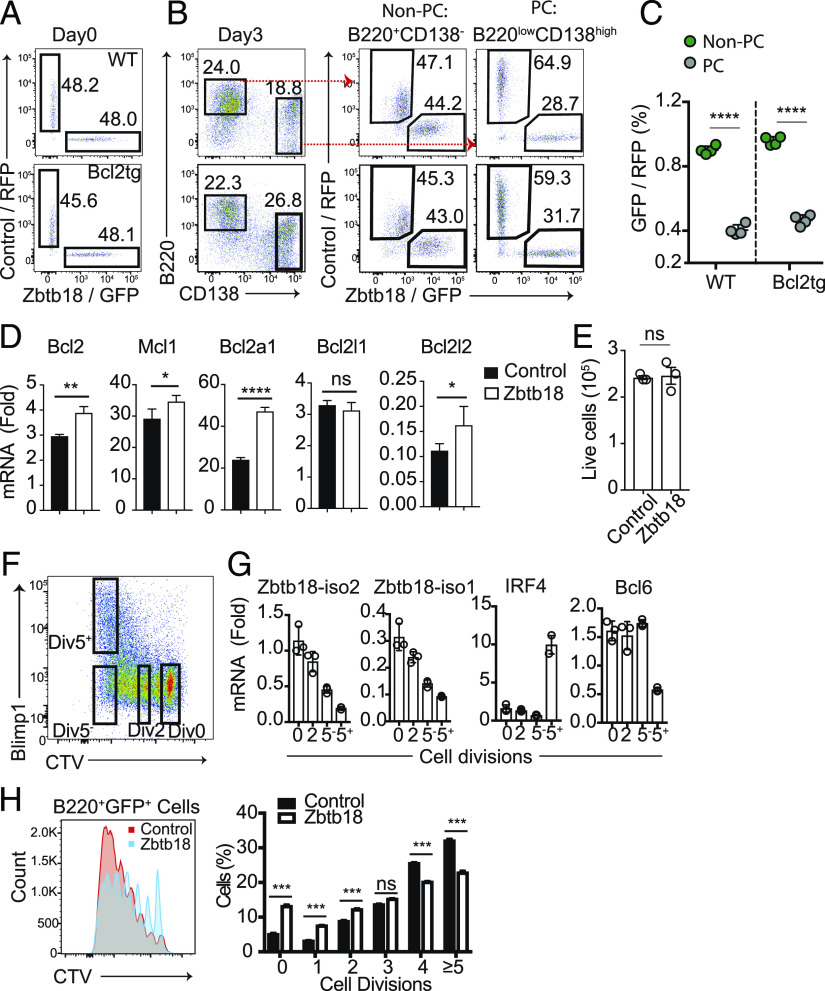
Zbtb18 regulates differentiation, but not survival of PCs. (**A**–**C**) Splenocytes derived from WT mice (upper panel) or Bcl2-Tg mice (lower panel) were transduced with control/RFP or Zbtb18/GFP retroviral vectors, sorted, and incubated with anti-CD40 and IL-4, as described in Fig. 1A. Cocultured cells were analyzed by flow cytometry before (A) and 3 d (B) poststimulation. The relative frequencies of GFP^+^ and RFP^+^ cells in the non-PC and PCs compartments are shown (C). (A)–(C) show the results of one representative experiment out of three independent experiments performed. Each circle represents a technical replicate. (**D** and **E**) Control/GFP– or Zbtb18/GFP–transduced splenocytes were incubated with anti-CD40 and IL-4. After 3 d, cells were sorted (GFP^+^ B220^+^ live cells) and subjected to qPCR analysis of antiapoptotic genes (D). Expression of mRNA is presented relative to the abundance of GAPDH (*n* = 3). Live count (trypan blue) was performed to determine viability (E). Data in (D) and (E) show the results from one representative experiment out of three independent experiments performed. Each circle represents a technical replicate. (**F** and **G**) Cells derived from Blimp-1–Venus–transgenic mice were labeled with the cell tracer dye CTV and stimulated with IL-4 and anti-CD40 for 3 d. Cells were sorted based on CTV dilutions (divisions [Div] 0, 2, and 5) and Blimp-1 expression and each fraction was analyzed by qPCR for expression of the indicated genes. Left, Flow cytometry showing sorting strategy to purify dividing cells. Right, Expression of mRNA is presented relative to the abundance of GAPDH and HPRT. Data shown in (F) and (G) are from one representative experiment out of three independent experiments performed. Each circle represents one technical replicate. (**H**) Cell division and PC differentiation in CTV-labeled splenic B cells from BM chimeras transduced with control/GFP or Zbtb18/GFP retroviral vector followed by 3 d incubation with anti-CD40 and IL-4. Left, Representative flow cytometry plots showing cell division. Cells were pregated on GFP^+^ B220^+^ cells. Right, Frequencies of cells in each cell division of total B cells. Data shown in (H) are from one representative experiment out of three independent experiments performed (*n* = 3, technical replicates). *p < 0.05, **p < 0.01, ***p < 0.001, ****p < 0.0001.

Zbtb18 may reduce PC responses by negatively regulating their survival. To address this possibility, we asked whether enhanced expression of the antiapoptotic regulator Bcl-2 can rescue Zbtb18-mediated suppression of PCs. We expect that if Zbtb18 limits PCs numbers because of impaired survival, ectopic expression of Bcl-2 (which negates key apoptotic pathways in B cells) (17) will reduce this effect. Zbtb18- or control-expressing vectors were transduced in splenocytes derived from animals carrying the Bcl-2 transgene and were cocultured, stimulated, and analyzed as described in Fig. 1A. Although Bcl-2–expressing B cells showed increase in PC expansion compared with WT-stimulated cells, as expected, Zbtb18 overexpression led to a significant reduction in PC frequencies at a level similar to that observed in WT-transduced controls (Fig. 2A–C). Furthermore, Zbtb18 expression was not associated with reductions in mRNA levels of several Bcl-2 family members, including the key PC survival regulator Mcl1, as well as Bcl2a, Bcl2l1, and Bcl2l2 (17, 35, 36) (Fig. 2D), or with increased apoptosis rates (Fig. 2E).

PC differentiation has been tightly linked to cell proliferation (3, 6, 15). To investigate the kinetics of Zbtb18 expression during cell division, we labeled B cells from a Blimp-1–Venus^−^–transgenic mouse line with the CellTrace Violet (CTV) cell tracer dye, activated them, sorted cells from divisions 0, 2, and 5, and analyzed each fraction by qPCR for expression of *Zbtb18*, *Irf4*, and *Bcl6*. Cells that have upregulated the PC master regulator Blimp-1 (Blimp-1–Venus^+^) were also sorted and analyzed at the same time from division 5 (Fig. 2F). As previously reported (6), induction of *Irf4* correlated with the upregulation of Blimp-1, whereas Bcl6 expression dropped abruptly at this stage (Fig. 2G). In contrast, Zbtb18 expression was reduced gradually between divisions 0, 2, and 5, reaching its lowest levels in Blimp-1^+^ cells that divided more than five times. To ask whether downregulation of Zbtb18 is associated with rates of B cell proliferation, we generated chimeras reconstituted with BM cells transduced with Zbtb18 or control GFP vector. We did not detect any gross defects in the ability of transduced BM cells to mature and differentiate into B cell subsets in these mice (data not shown). Control or Zbtb18-expressing B cells were labeled with a cell tracer dye and stimulated to induce PCs in vitro. Flow cytometry analysis revealed that, in Zbtb18-overexpressing cells, rates of cell division were reduced (Fig. 2H). Thus, these results show that downregulation of Zbtb18 correlates with gradual increase in PC differentiation. Notably, although constitutive expression of Zbtb18 inhibited PC responses, partial inhibition of this protein using shRNA approach did not lead to spontaneous activation of the cells or to enhanced PC responses (Supplemental Fig. 2D, 2E). This observation suggests that Zbtb18 likely contributes to mechanisms that inhibit cell activation and that, although this function is largely redundant under steady-state conditions (possibly because of the presence of other dominant factors that target the same pathway in resting B cells), downregulation of Zbtb18 is necessary to release the cells from this inhibition and allow PC differentiation to progress.

### Zbtb18 modulates chromatin accessibility of the PI3K pathway

**FIGURE 3.**
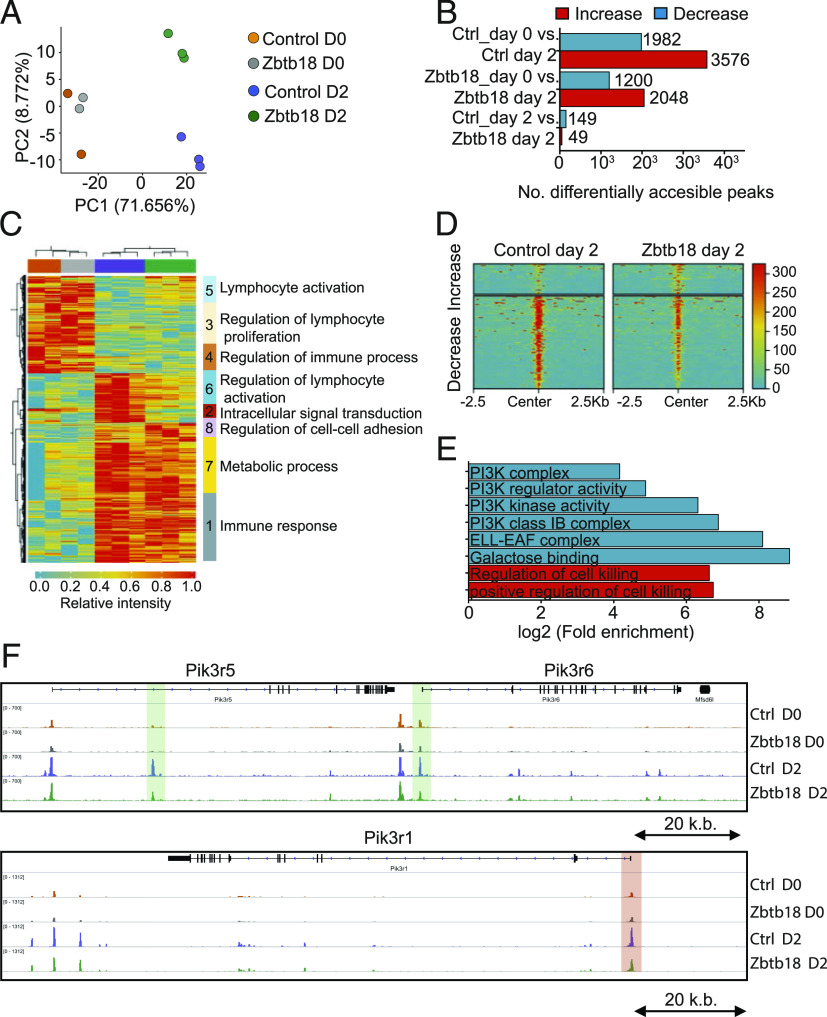
The chromatin landscape of activated B cell is modified by Zbtb18. (**A**) Principal components 1 and 2 (PC1 and PC2) ANOVA stabilized (VST) counts at differentially accessible peaks (FDR < 0.05) in control (RFP^+^ B220^+^ CD138^−^ cells) and Zbtb18-overexpression groups (GFP^+^ B220^+^ CD138^−^ cells) sorted by flow cytometry. Transduced cells were sorted on day 0 and day 2 after being cocultured in the presence of anti-CD40 and IL-4. (**B**) Number of differentially expressed genes at day 0 and 2 of B cells transduced with control or Zbtb18. (**C**) Heatmap of differentially accessible peaks color coded as in (A). Data are row scaled, and hierarchical clustering is based on Manhattan distances. (**D**) Heatmaps (top) of ATAC-seq read coverage (fragments per kilobase per million [FPKM]) at a 5-kb window centered over differentially accessible peaks. (**E**) Gene ontology analysis of genes that lose (blue) or gain (red) accessibility after Zbtb18 overexpression on day 2. (**F**) Normalized ATAC-seq coverage tracks (FPKM) for representative genomic regions at *Pik3r5*, *Pik3r6*, and *Pik3r1*, differentially accessible peaks highlighted by shading. Read density tracks from pooled replicates of one experiment performed (*n* = 2 for day 0, *n* = 3 for day 2).

To identify molecular mechanisms of Zbtb18 function, we explored the effect of Zbtb18 on chromatin accessibility in activated B cells using ATAC-seq. We hypothesized that enforcing constitutive expression of Zbtb18 will allow us to identify specific loci that fail to open shortly after activation, thus revealing potential targets. Because we found that Zbtb18 is rapidly downregulated after stimulation (Fig. 2F), we focused our analysis on the early phase of activation (day 2). Transduced B cells were sorted (GFP^+^ B220^+^ CD138^−^) and analyzed by ATAC-seq. For baseline control, transduced cells were similarly sorted prior to stimulation (day 0). Principal component analysis of differentially accessible peaks detected across all samples indicated that the main source of variation was between cells that received anti-CD40 and IL-4 (day 2) and those that did not receive this stimulation (day 0), regardless of Zbtb18 expression (Fig. 3A). This is further reflected by the large number of differentially accessible peaks between day 0 and day 2 in both control- and Zbtb18-overexpressing conditions. However, the second major variation, principal component-2, accounts for specific changes induced by constitutive expression of Zbtb18 (Fig. 3A) with relatively few peaks modified by Zbtb18 2 d poststimulation (Fig. 3B), indicating that Zbtb18 acts to regulate a restricted set of target loci.

Changes induced by constitutive Zbtb18 expression were most prominent on day 2 after stimulation with IL-4 and anti-CD40, consistent with the notion that Zbtb18 is expressed in resting cells and therefore its overexpression is expected to mostly affect cells after robust activation that promotes PC development (such as anti-CD40 and IL-4). Hierarchical clustering of 9105 differentially accessible peaks (FDR < 0.05) identified eight unique clusters (Fig. 3C). Genes associated with activation induced gains in accessibility (clusters 1 and 7) were enriched with immune response (e.g., *CD24a*, *Bcl6*, *Ripk2*, *Foxp1*: cluster 1) and metabolic processes (e.g., *Cox8a*, *Ndufab1*, *Pgk1*, *Ppif*: cluster 7) genes, whereas decreases in accessibility (clusters 4 and 3) were associated with genes encoding for immune system process (e.g., *Cd28*, *Cxcr4*, *Itgav*, *Irf4*: cluster 4) and regulation of lymphocyte proliferation (e.g., *Bcl2*, *Cd44*, *Hes1*, *Il21*: cluster 3). Overall, we identified 5558 peaks that were modified during differentiation (in control cells, fold change > 1; FDR < 0.05), with 3576 peaks increasing in accessibility from day 0 to day 2, and 1982 peaks decreasing. These observations are in agreement with a recent publication in which global changes in the chromatin landscape during the early phase of PC differentiation were described (15). Notably, we also identified differentially accessible genes that were specifically induced by constitutive expression of Zbtb18, including some loci that showed enhanced accessibility (clusters 5 and 8 [e.g., *Asmt*, *Trpc1*, *Plcd1*, *Cdk17*, *Myc*, *Syk*]) and some in which reduction in chromatin open structures was observed (clusters 2 and 6 [e.g., *Ufm1*, *Smad7*, *Srgn*, *Asah2*, *Mxd4*, *Tle3*]). Closer examination of the most significant changes in ATAC-seq peaks induced by Zbtb18 indicated that the majority were linked to decrease in chromatin accessibility (Fig. 3D). These findings are consistent with a role for Zbtb18 as a transcription corepressor.

**FIGURE 4.**
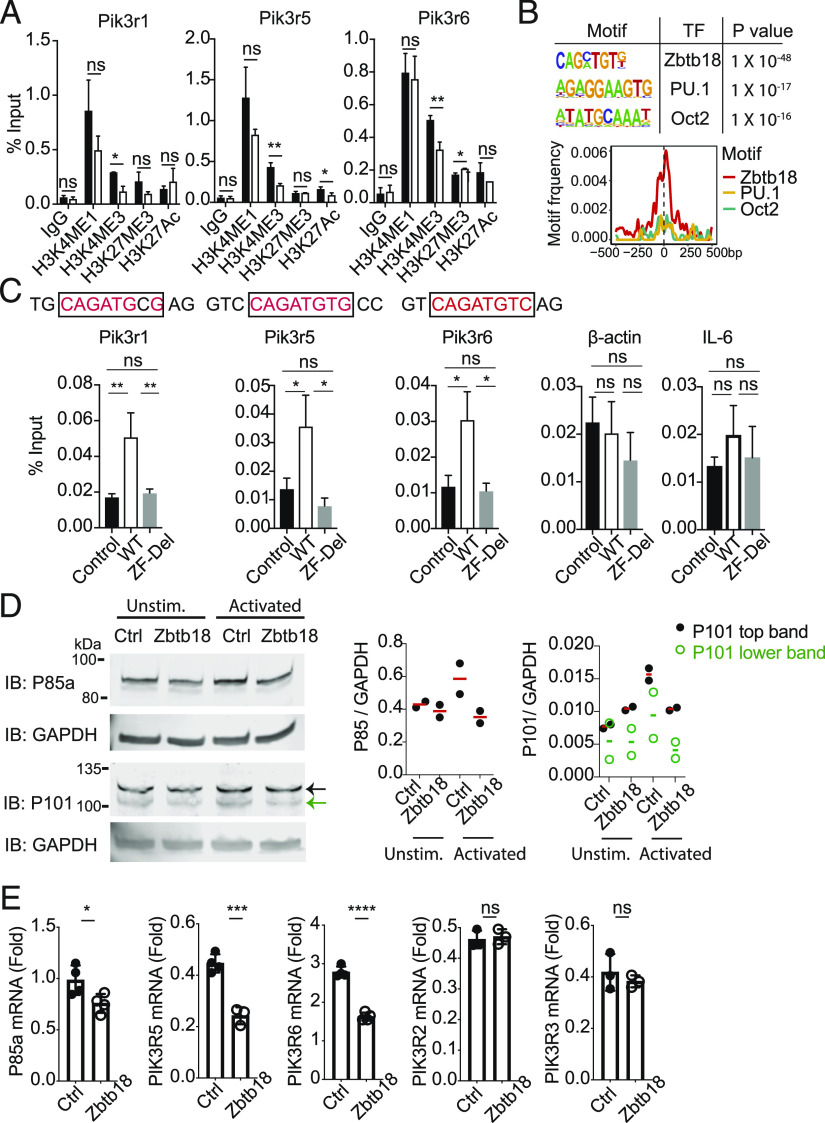
Zbtb18 directly binds and suppresses expression of PI3K class I genes. (**A**) Splenocytes were transduced with control (RFP^+^) or Zbtb18 (GFP^+^) vectors. Cells were analyzed by flow cytometry and mixed to achieve a 1:1 ratio of B220^+^-GFP^+^– and RFP^+^-expressing cells, followed by stimulation with anti-CD40 and IL-4. Two days after stimulation, RFP^+^ or GFP^+^ B220^+^ CD138^–^ live cells were sorted by flow cytometry and subjected to Chip qPCR analysis of the ATAC-seq differential peaks of *Pik3r1*, *Pik3r5*, and *Pik3r6* genes. Data are from pooled samples from three independent experiments performed (total *n* = 3). (**B**) De novo motif–enrichment analysis in repressed ATAC-seq peaks of Zbtb18 versus control (Ctrl) cells. Motif density distribution relative to the peak summit for Zbtb18, Pu.1 and Oct2 motifs in the repressed ATAC-seq peaks (bottom). (**C**) Chip qPCR analysis of interactions between Myc-tagged Zbtb18 (WT), or ΔZF with the indicated genes. Ctrl B cells transduced with empty GFP-expressing vector. Conserved Zbtb18-binding motif identified in the ATAC-seq peaks of the indicated genes are shown in red. Analysis was performed on transduced sorted cells (live B220^+^ GFP^+^ cells) 2 d after anti-CD40 and IL-4 stimulation. Data show pooled samples collected in three independent experiments performed (total *n* = 3, biological replicates). (**D**) Immunoblot analysis of p85a, p101, and GAPDH in Ctrl/RFP– or Zbtb18/GFP–transduced B cells 2 d after being cocultured with or without anti-CD40 and IL4 stimulation (left) [as in (A) above]. Cells were sorted prior to analysis on day 2 (gated on live B220^+^ CD138^−^ GFP^+^ or RFP^+^ cells). Quantification of p85a or p101 expression normalized with GAPDH (right). Figure shows combined data collected in two independent experiments performed. Each circle represents data from one experiment. (**E**) qPCR analysis of the indicated genes in control- and Zbtb18-transduced B cells, sorted (as GFP^+^ B220^+^ CD138^−^ live cells) 2 d poststimulation with anti-CD40 and IL-4. Expression of mRNA is presented relative to the abundance of GAPDH. Data in (E) show the results from one representative experiment out of three independent experiments performed. Each circle represents a technical replicate. *p < 0.05, **p < 0.01, ***p < 0.001, ****p < 0.0001.

Importantly, among the Zbtb18-induced chromatin-remodeled loci, genes belonging to the class I phosphoinositide 3-kinase (P13K) pathway were significantly (FDR < 0.05) enriched (Fig. 3E). The most prominent changes were detected in the ATAC-seq peaks of two genes encoding for regulatory subunits of the PI3K catalytic subunit p110γ. These included *Pik3r5* and *Pik3r6*, which encode for p101 and p87, respectively. In these genomic regions, stimulation-induced opening of chromatin structures was strongly inhibited by Zbtb18 (Fig. 3F). This included a 1.9-fold reduction in the peak height of *Pik3r5* (adjusted *p* value of 1.58 × 10^−06^), and a 1.6-fold reduction in the peak of *Pik3r6* (adjusted *p* value of 0.0024). A trend indicating suppression of chromatin opening by Zbtb18 overexpression was also observed at *Pi3kr1*, (a gene encoding for the p85α regulatory subunit of p110δ) (Fig. 3F), *Pi3kcd*, and *Pik3cg* peaks (Supplemental Fig. 3A); although these observations did not reach the significance threshold. In contrast, the ATAC-seq peaks of β-actin and IL-6 were unaffected by overexpression of Zbtb18 on days 0 or 2 (Supplemental Fig. 3B). To validate these results, we next employed Chip assay followed by qPCR analysis in control- and Zbtb18-transduced activated B cells. This analysis showed that Zbtb18 significantly suppressed association of H3K4Me3 with the above genes, whereas other markers for open chromatin structure, including H3K27me3 and H3K27Ac, showed more variable effects suppressing some, but not all, loci (Fig. 4A, Supplemental Fig. 3C). As expected, β-actin and IL-6 loci, which are not regulated by Zbtb18, showed similar levels of accessibility in control- versus Zbtb18-transduced B cells (Supplemental Fig. 3C). Together, these results support a role for Zbtb18 as a transcriptional suppressor of the PI3K pathway.

### The zinc finger domain of Zbtb18 directly interacts with its target DNA motif to regulate B cell differentiation

Zinc finger domains are known to bind their DNA targets via defined motifs. We, therefore, next used our ATAC-seq data to perform de novo (transcription factor–binding) motif–enrichment analysis in genes that were suppressed by Zbtb18 overexpression. Zbtb18-binding motifs were significantly enriched in the repressed ATAC-seq peaks in Zbtb18-transduced B cells compared with the control group at day 2 (Fig. 4B). A moderate but significant enrichment of the binding motifs of two other B cell response regulators including PU.1 (37, 38) and Oct2 (39, 40) was also noted (Fig. 4B), possibly suggesting collaborative or competing interactions with Zbtb18.

Importantly, several Zbtb18 motifs (41) were identified in the repressed peaks of *Pik3r1*, *Pik3r5*, and *Pi3kr6* (Fig. 4C, highlighted in red), supporting the possibility that Zbtb18 directly binds to these genes. In agreement, Chip qPCR analysis in B cells transduced with an Myc-tagged Zbtb18 showed a clear and selective interaction with DNA elements in *Pik3r1*, *Pik3r5*, and *Pi3kr6*, but not in control genes including β-actin or IL-6 (Fig. 4C). Furthermore, this interaction was lost in B cells transduced with the Zbtb18 zinc finger–deficient mutant (ΔZF), supporting a critical role for this domain in binding DNA motifs of class I PI3K regulatory genes (Fig. 4C). Consistent with loss of binding to PI3K genes, the truncated ΔZF Zbtb18 also lost its ability to suppress PC responses (Supplemental Fig. 4A–C). We, therefore, propose that downregulation of Zbtb18 during activation may allow dividing B cells to gradually lose Zbtb18-mediated inhibition and increase PI3K activity and that this effect may contribute to enhanced differentiation of PCs.

### Downregulation of Zbtb18 modulates PI3K activity in stimulated B cells

The above hypothesis suggests that following activation B cells will increase the total amount of PI3K subunits in the cell and that downregulation of Zbtb18 will be necessary for this process. To test this possibility, we assessed protein levels of PI3K subunits in B cells transduced with control or Zbtb18 retroviral vector before and after stimulation. Within 2 d poststimulation with IL-4 and anti-CD40, we noted an increase in the total protein level of PIK3R1 (P85) and PIK3R5 (P101) in control-transduced cells (Fig. 4D), supporting the hypothesis that expression of PI3K genes is enhanced during the early phase of activation. Notably, in addition to the expected PIK3R5 band size of ∼100 kDa (green arrow Fig. 4D), we also detected a slightly higher specific band at ∼115 kDa (black arrow), which displayed a similar trend. PIK3R6 was excluded from the analysis because, despite our efforts, we could not clearly detect it by Western blot, possibly because of its relatively low expression levels in hematopoietic cells (42). The finding that levels of PIK3R1 and PIK3R5 were only mildly increased in control-activated cells could partially be because of the relatively early time point we chose for analysis, at a time when PI3K proteins may not yet accumulated in the cells. Attempts to explore later timepoints were unsuccessful because many transduced B cells that did not receive anti-CD40 and IL-4 stimulation died within 3 d in tissue culture, thus preventing direct comparison with baseline levels. Importantly, however, B cells in which Zbtb18 was constitutively expressed did not show detectable changes in PI3KR1 and PI3KR5 expression upon activation (Fig. 4D), supporting the possibility that downregulation of Zbtb18 shortly after stimulation is needed to release transcriptional repression of PI3K genes. In line with this hypothesis, mRNA levels of PI3KR1, PI3KR5, and PI3KR6 were significantly and specifically reduced in Zbtb18-overexpressing B cells, as determined 2 d poststimulation with IL-4 and anti-CD40 (Fig. 4E).

**FIGURE 5.**
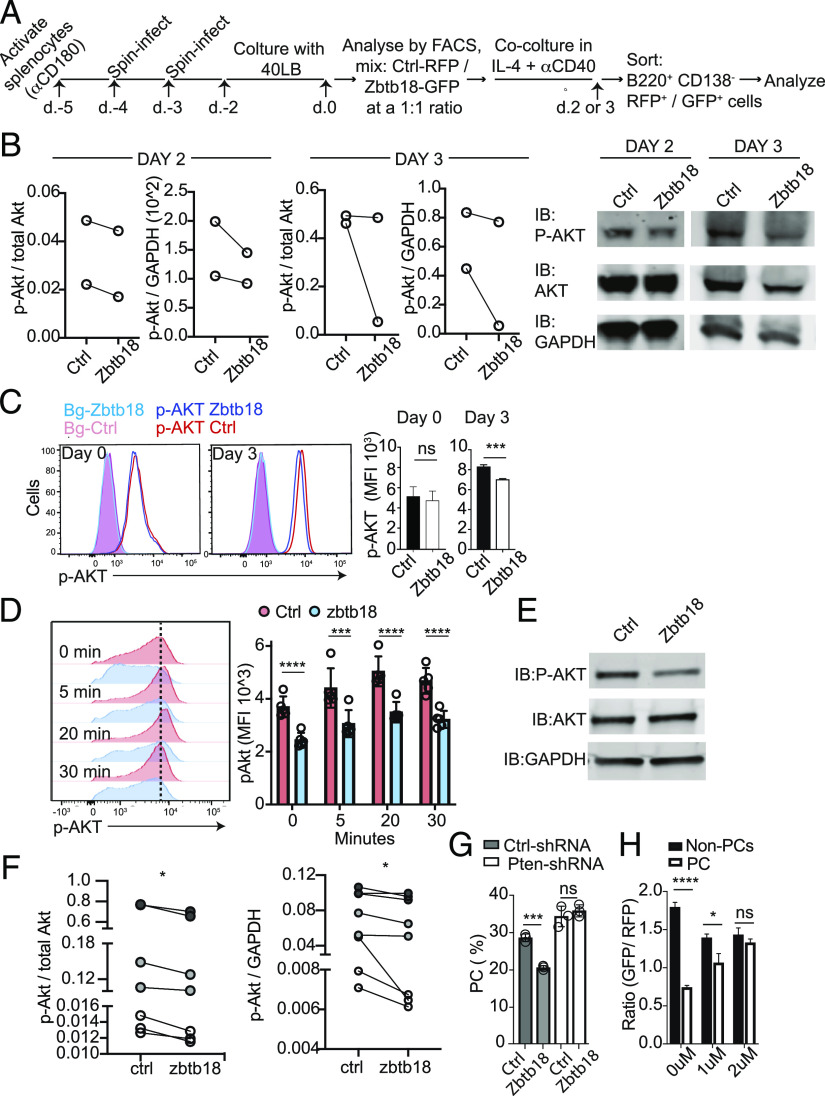
Zbtb18 overexpression negatively regulates PI3K signaling in B cells. (**A** and **B**) Immunoblot analysis of p-Akt, total Akt, and GAPDH in Ctrl/RFP– or Zbtb18/GFP–transduced activated B cells. The schematic of the experiment is shown in (A). Left, Quantification of p-Akt normalized against total Akt and GAPDH. Figure shows data from four independent experiments performed. Each circle represents data from one mouse transduced and stimulated independently. Right, A representative immunoblots from the above. (**C**) Intracellular stain of p-AKT in Ctrl/RFP– or Zbtb18/GFP–transduced B cells before (day 0) or 3 d post coincubation in the presence IL-4 and anti-CD40. Histograms are pregated on B220^+^ CD138^−^ cells. Cells were preincubated with 40LB prior to stimulation and mixed, as in (A). Left, A representative flow cytometry plot. Right, Average MFI of p-AKT from technical replicates (*n* = 3). The figure shows the result from one independent experiment out of four performed. (**D**) WEHI cells were stably transduced with control/RFP and Zbtb18/GFP and cocultured for 2 d. Cells were stimulated with anti-IgM and anti-CD40 for the indicated time points, fixed, and stained for intracellular p-Akt. Left, A representative histogram pregated on RFP^+^ (control)- or GFP^+^ (Zbtb18)-transduced cells. Right, quantification of average p-Akt MFI in technical replicates (*n* = 4) from one representative experiment out of three performed. (**E** and **F**) WEHI cells stably transduced with control/RFP and Zbtb18/GFP were cocultured for 2 d. RFP^+^ and GFP^+^ cells were sorted and subjected to immunoblot analysis to detect levels of p-Akt, total Akt, and GAPDH. A representative immunoblots is shown in (E). In (F), quantification of p-Akt normalized against total Akt and GAPDH. (F) shows pooled data from three independent experiments (color coded). One circle represents data from one biological replicate. (**G**) Control/GFP– or Zbtb18/GFP–transduced splenocytes were cotransduced with control-shRNA/RFP or PTEN-shRNA/RFP, as indicated. Cells were stimulated for 3 d posttransduction with anti-CD40 and IL-4. Frequencies of PCs in GFP^+^ RFP^+^ cotransduced B cells were determined by flow cytometry. The figure shows one out of three independent experiments performed. Each circle represents one technical replicate. (**H**) B cells transduced with control/RFP or Zbtb18/GFP were sorted, cocultured, and incubated with LPS with or without increasing concentrations of FOXO1 inhibitor and analyzed by flow cytometry 3 d later. The frequencies of GFP^+^ and RFP^+^ cells in the non-PCs (B220^high^ CD138^−^) and PCs (B220^low^ CD138^high^) populations are shown. Figure shows one out of three independent experiments performed (*n* = 3, technical replicates). *p < 0.05, ***p < 0.001, ****p < 0.0001.

Although the above findings show that Zbtb18 directly suppresses expression of class I PI3K subunits, they do not directly confirm that this effect has a measurable quantitative impact on PI3K signaling or PC differentiation. To next test this possibility, we assessed AKT phosphorylation levels in activated Zbtb18-overexpressing B cells by Western blot analysis. In these experiments we preincubated the transduced cells on the 40LB cell line to enhance survival and activation (experimental setup shown in Fig. 5A). In agreement with a role for Zbtb18 in inhibiting PI3K signals, Zbtb18-overexpressing B cells displayed a modest but consistent reduction in AKT phosphorylation (Fig. 5B). Similar results were obtained when p-AKT levels were tested using flow cytometry analysis (Fig. 5C). As a complementary approach to further test the effect of Zbtb18 expression on PI3K signaling, we stably transduced the B cell line WEHI-231 with Zbtb18/GFP or control/RFP vector. The transuded cells were coincubated and stimulated with anti-IgM and anti-CD40 for 5, 20, and 30 min. Flow cytometry analysis revealed that in Zbtb18-overexpressing cells, the mean fluorescence intensity (MFI) of p-AKT was reduced by 30–35% compared with the control group (Fig. 5D), supporting a role for Zbtb18 in limiting PI3K signals in these cells. Of note, this effect was already evident in unstimulated WEHIs, possibly reflecting a higher baseline AKT activity in these cells. Consistent with these results, Western blot analysis showed a similar trend of reduced p-AKT levels in Zbtb18-transduced WEHIs compared with controls (Fig. 5E, 5F).

Given the importance of PI3K signaling for PC differentiation (7, 8, 10, 11), we hypothesized that the defects observed in PC differentiation of overexpressing Zbtb18 cells may be partially explained by loss of their ability to enhance PI3K signals. To test this possibility, we asked whether enhancing PI3K signaling by inhibiting negative regulators of the PI3K pathway will overcome Zbtb18-mediated suppression. PI3K activity is negated by PTEN, a constitutively expressed phosphatase that terminates class I PI3K signaling by dephosphorylating PIP_3_ into PI(4,5)P_2_. To test whether inhibiting PTEN will overcome ZBtb18-mediated PC suppression, we cotransduced B cells with Zbtb18 or control vector together with PTEN-shRNA or control shRNA. The efficiency of the PTEN-shRNA was confirmed by qPCR and in vitro PC differentiation assay (Supplemental Fig. 4D, 4E). Cotransduced cells were stimulated with IL-4 and anti-CD40 for 3 d and analyzed by flow cytometry. As expected, cotransduction of cells with Zbtb18 and control shRNA led to ∼30% reduction in PC development compared with cells coexpressing the control vector and control shRNA (Fig. 5G). However, when PTEN was silenced, PC frequencies were increased and Zbtb18 constitutive expression no longer inhibited the response (Fig. 5G). Moreover, direct inhibition of FOXO1, one of the major cellular targets that are inactivated by the PI3K pathway in B cells, effectively negated Zbtb18-mediated suppression of PC differentiation and restored the response (Fig. 5H). Together, these observations are consistent with the notion that Zbtb18-mediated suppression of PC differentiation is at least partially facilitated by its transcriptional repression of PI3K genes.

### Zbtb18 suppresses differentiation of human PCs

**FIGURE 6.**
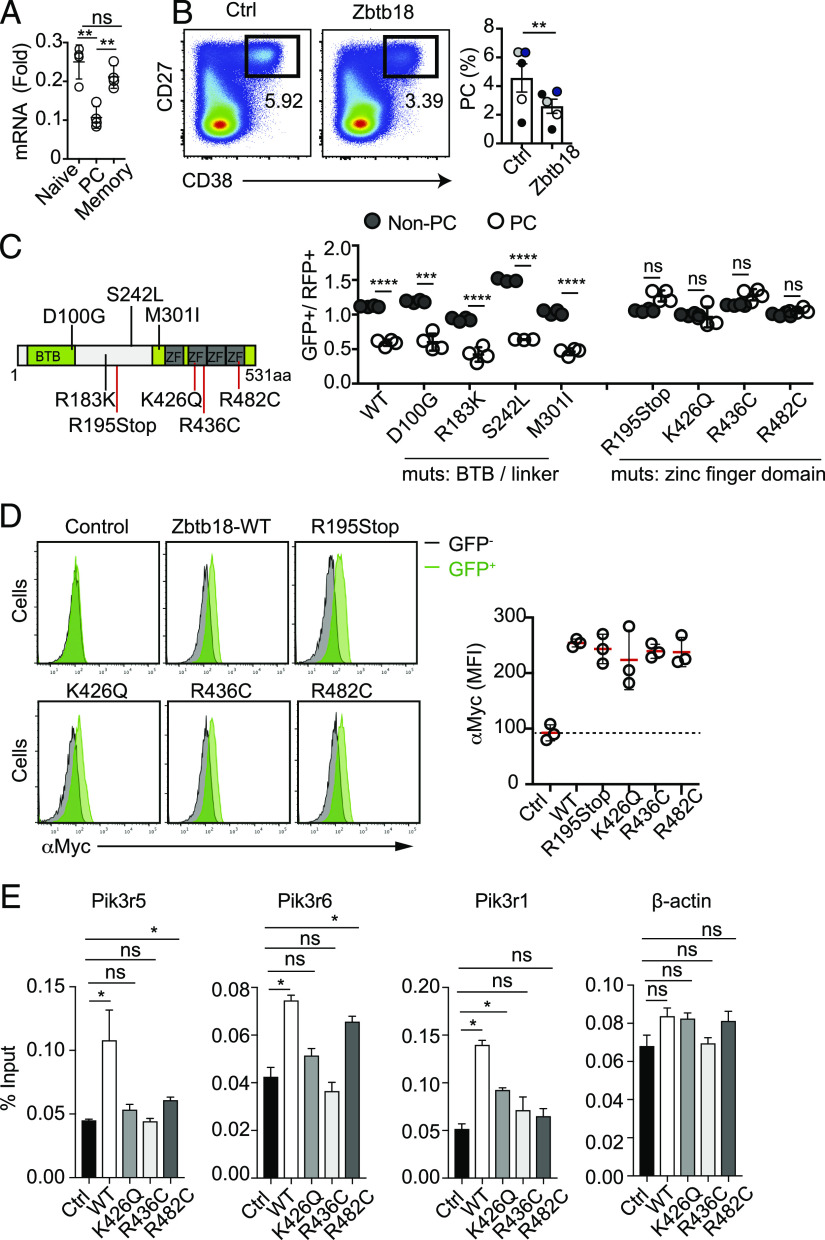
Zbtb18 suppresses differentiation of human PCs. (**A**) qPCR analysis of the transcript abundance of Zbtb18 in human naive cell (CD19^+^IgD^+^CD27^−^), memory cell (CD19^+^ CD27^+^ CD38^−^), and PC (CD19^+^IgD^−^CD27^+^CD38^+^) sorted from human peripheral blood samples. Expression of mRNA is presented relative to the abundance of RPL13A. Data are from two biological replicates analyzed with two technical replicates each. The experiment is one out of two independent experiments performed. (**B**) Human B cells were purified from healthy donors, stimulated, and electroporated with control (Ctrl) or Zbtb18 RNA. Left, Representative example of flow cytometry analysis of cells 5 d poststimulation pregated on CD19^+^cells. Right, Frequencies of PCs (CD38^+^ CD27^+^). Data are from pooled samples from four independent experiments performed. Each experiment is color coded. (**C**) Left, Schematic illustration of point mutations in Zbtb18 identified in cancer patients. Red lines highlight mutations that target residues within the zinc finger motif or that lead to loss of the entire domain. Right, B cells from mouse spleen were transduced with control/RFP or Zbtb18-Myc–Tagged/GFP WT or mutants, sorted, coincubated, and stimulated with IL-4 and anti-CD40 for 3 d (as in Fig. 1A). Shown are the frequencies of non-PC and PC populations in GFP- and RFP-expressing cells. Data show one representative experiment out of three independent experiments performed. Each circle represents a technical replicate. (**D**) Anti-Myc intracellular stain splenocytes transduced with Zbtb18-Myc–tagged WT and mutants. Ctrl B cells transduced with empty (GFP-only) vector. Histograms are pregated on untransduced (GFP^−^, black) and transduced (GFP^+^, green) fractions. Left, Representative histograms. Right, MFIs of Myc. Figure shows data from one representative experiment out of three independent experiments performed. Each circle represents a technical replicate. (**E**) Chip qPCR analysis for binding of Zbtb18-Myc (WT) or the indicated mutated forms to class I PI3K genes. β-actin was used as a nonspecific binding gene. Ctrl B cells transduced with empty (GFP) vector. Analysis was performed on sorted cells (live B220^+^ GFP^+^) 2 d after anti-CD40 and IL-4 stimulation. Figure shows one representative experiment out of three independent experiments performed (*n* = 4, technical replicates). *p < 0.05, **p < 0.01, ****p < 0.0001.

The mouse Zbtb18 protein shares 99% homology with its human counterpart (43). We next examined expression pattern of ZBTB18 in human B cells purified from the blood of healthy donors. Similar in mice, we find that expression of ZBTB18 was significantly downregulated in PCs compared with naive and memory human B cells (Fig. 6A). To assess whether ZBTB18 also functions similarly in human B cells and suppresses PC development, we enforced expression of ZBTB18 mRNA in purified stimulated human B cells and analyzed the relative frequencies of PCs. A GFP-expressing vector was used as control. mRNA was electroporated into purified B cells 2 d after their culture in the presence or absence of stimulation. We hypothesized that, at this time point, endogenous Zbtb18 begins to decline and, therefore, its enforced expression at this stage may interfere with the ability of cells to progress toward PC fate. In agreement with this hypothesis, we found that cells that received Zbtb18 RNA showed a significant and consistent reduction in PC formation (Fig. 6B), suggesting Zbtb18 has a similar PC suppression capacity in human B cells.

Somatic mutations of Zbtb18 have been identified in diseases associated with excessive activity of PI3K, including several human cancers including glioblastoma, ovary, gut, and acute lymphoblastic leukemia patients (44–57). To gain additional insight into mechanisms of Zbtb18 activity, we transduced B cells with mutated forms of ZBTB18 (Myc-tagged) derived from such patients and tested their ability to suppress PC differentiation. Among the eight mutants tested, we find that all those in which the zinc finger motif was interrupted (including R195-stop, K426Q, R436C, and R482C) had lost the capacity to suppress PCs formation, indicating loss of ZBTB18 activity (Fig. 6C). Importantly, loss of activity was not due to failure to express the mutated protein or to localize it to the nucleus, as confirmed by flow cytometry analysis of Myc expression, immunofluorescent, and Western blot analysis (Fig. 6D, Supplemental Fig. 4F, 4G). Furthermore, Chip qPCR analysis confirmed impaired interactions of the mutants with *pi3kr1*, *pi3kr5*, and *pi3kr6* genes (Fig. 6E), indicating that, in addition to loss of activity in suppressing PC responses, these ZBTB18-mutated proteins are dysfunctional in their capacity to regulate PI3K signaling.

## Discussion

PC differentiation is an irreversible process that involves a massive shift in the expression of hundreds of genes. This dramatic transition is mediated by a series of cell divisions during which the cells progressively lose their B cell identity and acquire PC fate (4–6). Previous studies have shown that during the early phase of PC differentiation, chromatin accessibility of class I PI3K genes increases (15). However, the mechanistic link between cell division and epigenetic regulation of this signaling pathway and the relevance of this process to PC differentiation have not been explored. In this study, we demonstrate that PC differentiation is associated with downregulation of Zbtb18, a ZFP, that we identify as a transcriptional repressor of class I PI3K regulatory subunits. We propose that cell division–coupled amplification of PI3K signals is promoted by gradual decrease in Zbtb18 and that this process plays a role in promoting PC differentiation.

Several lines of evidence support this conclusion. Using ATAC-seq analysis, we show that constitutive expression of Zbtb18 in activated B cells leads to decrease in chromatin accessibility of genes encoding for class I PI3K regulatory subunits, most notably of *Pik3r1*, *Pik3r5*, and *Pik3r6*. This was accompanied by lower association of the activating epigenetic marker H3K4Me3 with promoter/enhancer elements and with lower expression of PI3K proteins in activated B cells. Furthermore, we show that Zbtb18-binding motifs are enriched within DNA regions of several class I PI3K genes, most notably those encoding for *Pik3r1*, *Pik3r5*, and *Pik3r6*, and that Zbtb18 directly binds them. Collectively, these findings provide support to the notion that Zbtb18 acts as a transcriptional repressor that limits expression of PI3K regulatory subunits. Given the known role of PI3K signaling in driving PC responses, we propose that during the initial phase of B cell activation, downregulation of Zbtb18 is necessary to release transcriptional repression of PI3K genes consequently amplifying PI3K signals and enhancing PC differentiation. In line with this possibility, Zbtb18 overexpression in primary B cells or in the WEHI-231 B cell line was associated with recued AKT phosphorylation. Moreover, restoring proximal and distal activation of the PI3K signaling cascade negated Zbtb18-mediated inhibition and rescued PC differentiation.

Our results indicate that Zbtb18 inhibits, but not fully block, the PI3K pathway in B cells. This is evident by the fact that overexpression of Zbtb18 reduced but did not completely prevent PC formation. Furthermore, partial inhibition of Zbtb18 using shRNA approach did not lead to spontaneous or enhanced PC differentiation, suggesting that loss of Zbtb18 is not enough to drive activation. It is, therefore, likely that the ability of Zbtb18 to modulate PI3K activity in B cells is limited to defined differentiation steps where cells are either more sensitive to relatively subtle changes in PI3K signals or where alternative mechanisms that negatively regulate PI3K activity are downregulated. This may also explain why enforced expression of Zbtb18 did not have a significant effect on GC responses, which depend on PI3K signaling, possibly reflecting the differential manner by which PI3K pathway is regulated in these cells compared with naive B cells (22, 58, 59). We therefore propose that Zbtb18 functions as a rheostat to fine-tune B cell activation and that the extent of its effect is context dependent. Future studies using genetic mouse models to selectively and more efficiently delete Zbtb18 from B cells are necessary to further explore its role in regulating B cell homeostasis.

Triggering the PI3K signaling in B cells is known to negatively regulate class switch recombination (12, 60, 61). Surprisingly however, overexpression of Zbtb18 did not lead to impaired class switching. It is possible that the extent by which Zbtb18 modulates PI3K signals is insufficient to modify FOXO1-mediated control of CSR. In support of this possibility, FOXO1 heterozygous mice had normal levels of CSR (62). In addition, it is important to note that FOXO1 is not the only target of AKT and that PI3K also activates other effector proteins (63, 64). Thus, although our study show that Zbtb18 inhibits PCs at least partially by modulating the PI3K pathway and the FOXO1 activity, our findings do not rule out a contribution of other effector proteins downstream to PI3K to this effect or the existence of additional mechanisms by which Zbtb18 may modulate the differentiation of PCs.

The PI3K pathway plays a central role in regulating multiple cellular processes and is highly conserved among multicellular organisms. Dysregulation of PI3K activity is associated with many human diseases including cancer, diabetes, cardiovascular disorder, and neurologic diseases. Despite being of major relevance to human health, to date, little is known about the manner by which PI3K genes are transcriptionally regulated. Our finding that both mouse and human Zbtb18 has the capacity to bind class I PI3K genes and modulate their expression in B cells may have broader implications that extend beyond its role in regulating immune responses and that may be even more relevant to other cell types in which Zbtb18 is expressed. Notably, in humans, Zbtb18 is abundantly expressed in the brain where it has been shown to regulate the development of neuronal cells. Somatic mutations of Zbtb18 have been identified in various diseases, including human cancers (44–57). The role of Zbtb18 in these diseases is not well understood, but recent findings have shown that it may act as a tumor suppressor gene such that its loss may directly contribute to the development or progression of the disease (65–67). Given the importance of PI3K in regulation of multiple cellular processes and the tight association between excessive PI3K activity and cancer development, it will be important to determine whether Zbtb18 may play a more dominant role in regulating the PI3K pathways in other cell types and whether loss of its function may contribute to progression or onset of some diseases.

## Supplementary Material

Data Supplement
