# Supplementary material for: The Zinc Finger Protein Zbtb18 Represses Expression of Class I Phosphatidylinositol 3-Kinase Subunits and Inhibits Plasma Cell Differentiation
Source: J Immunol. 2021 Feb 19;206(7):1515–27. doi: 10.4049/jimmunol.2000367 (PMC7980533; doi:10.4049/jimmunol.2000367)
Supplement: Data Supplement [file JI_2000367.zip › JI_2000367_Supplemental_Figures_1.pdf]

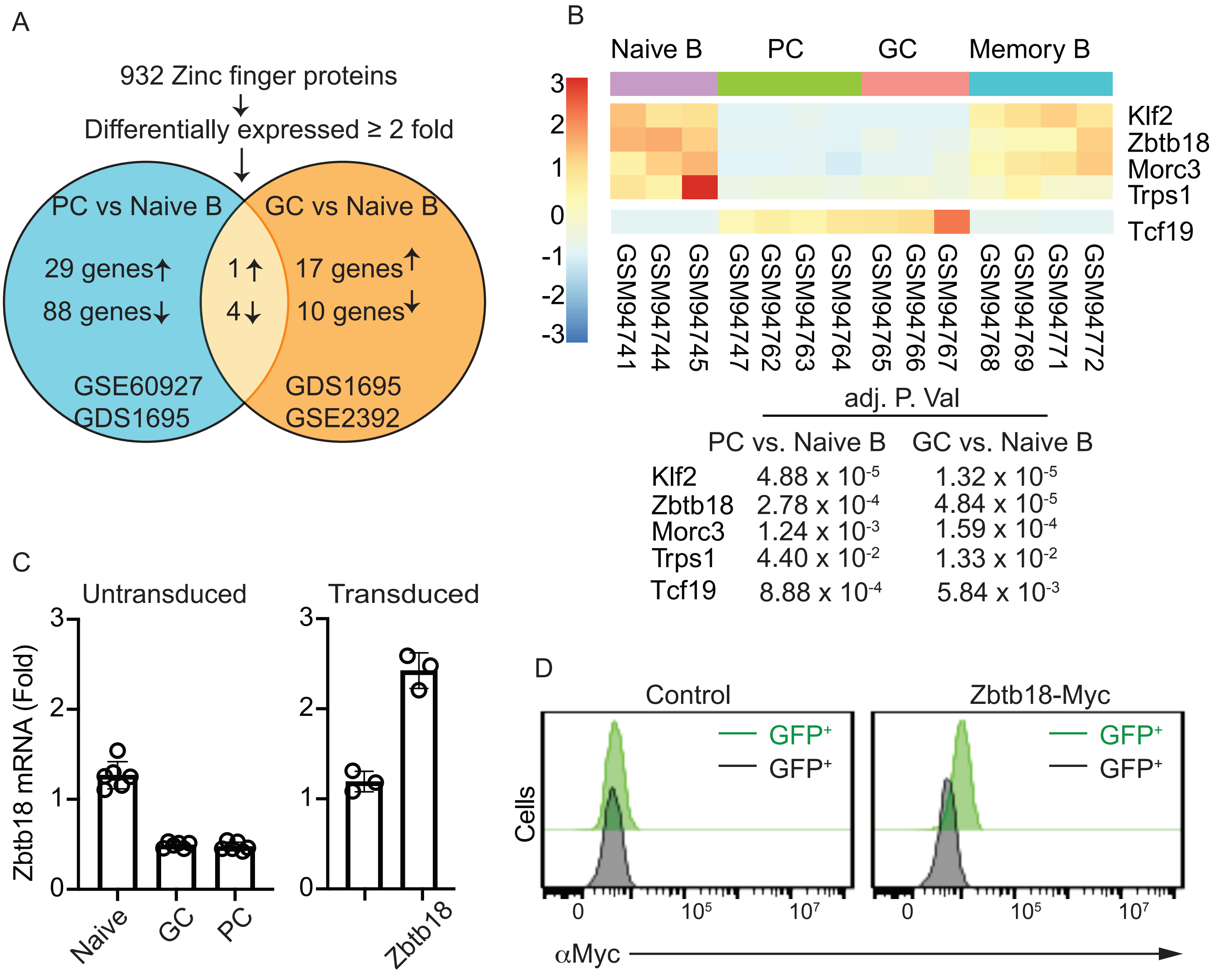

Supplementary figure 1. Zbtb18 is downregulated in activated B cells. (**A**) Strategy to identify Zinc finger proteins participating in humoral response from 3 online databases, GSE60927, GDS1695 and GSE2392. (**B**) Heap map showing relative expression levels of the top variably expressed 5 genes in GC B cells and PCs compared with naïve B and memory B cells. The 5 genes were ranked by adjusted P. values calculated (bottom). (**C**) Left, qPCR analysis of the transcript abundance of Zbtb18 in naïve (B220^{+} IgD^{high} FAS^{-} CD138^{-}), germinal centre (GC, B220^{+} IgD^{low}FAS^{high}GL7^{+}), plasma cells (PC, B220^{low} CD138^{+}) sorted from mice, 10 days post SRBC immunization. Right, qPCR analysis of Zbtb18 in B cells transduced with either control-RFP or Zbtb18-GFP. RFP^{+} and GFP^{+} B220^{+} cells were sorted prior to QPCR analysis. Data is displayed relatively to abundance of GAPDH. (**D**) Splenocytes were transduced with a retrovirus expressing GFP alone (control) or GFP and Zbtb18-Myc. Transduced cells were intracellularly stained for Myc expression in the GFP^{-} (black) and GFP^{+} (green) fractions. In panels C and D, the data represent one experiment out of 3 independent experiments performed. Each circle represents one technical replicate.

Figure S1

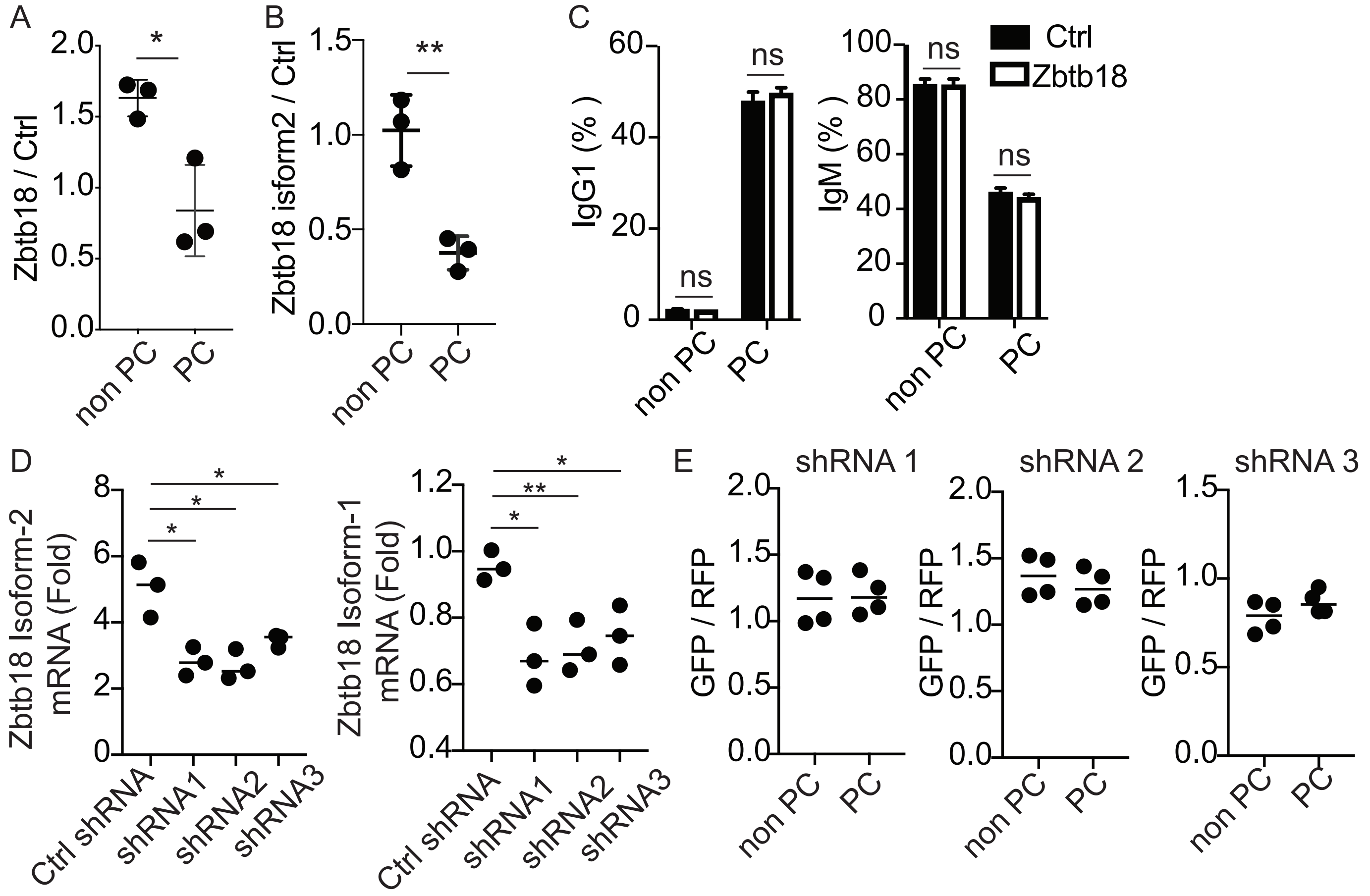

Supplementary figure 2. Zbtb18 suppresses PCs differentiation but not IgG1 class-switch recombination. (**A**) Splenocytes transduced with a Zbtb18/GFP or Control/RFP expressing vector were sorted as B220$^+$ GFP$^+$ or RFP$^+$ cells, co-incubated, stimulated with LPS and analysed by flow cytometry 3 days later to determine the relative frequencies of GFP$^+$ and RFP$^+$ cells in the non-PC and PCs compartments. (**B**) B cells transduced with Control/RFP and Zbtb18 spliced isoform-2/GFP were treated and analysed as above. (**C**) The percentage of IgG1$^+$ (left) and IgM$^+$ (right) cells in the non-PC and PC compartments in stimulated B cells transduced with Zbtb18 or control expressing vector (n=3, technical replicate). **(D)** Quantitative PCR analysis of the transcript abundance of isoforms 1 and 2 of Zbtb18 in that were transduced with either control-shRNA/RFP or different Zbtb18-shRNAs/GFP. Cells were sorted as B220$^+$ RFP$^+$ or GFP$^+$ prior to analysis. Expression of mRNA is presented relative to the abundance of GAPDH and HPRT. **(E)** B cells transduced with retrovirus expressing the Zbtb18-shRNA/GFP or control (scrambled)-ShRNA/RFP were sorted (as B220$^+$ RFP$^+$ or GFP$^+$ cells), mixed in a 1:1 ratio, incubated with anti-CD40 and IL4 for 3 days and analysed by flow cytometry. Frequencies of GFP$^+$ and RFP$^+$ cells in the Non-PC and PCs compartments on are shown. In each panel shown in A-E, the data represent one experiment out of 3 independent experiments performed. Each circle represents one technical replicate. In C, n=3 (technical replicates).

Figure S2

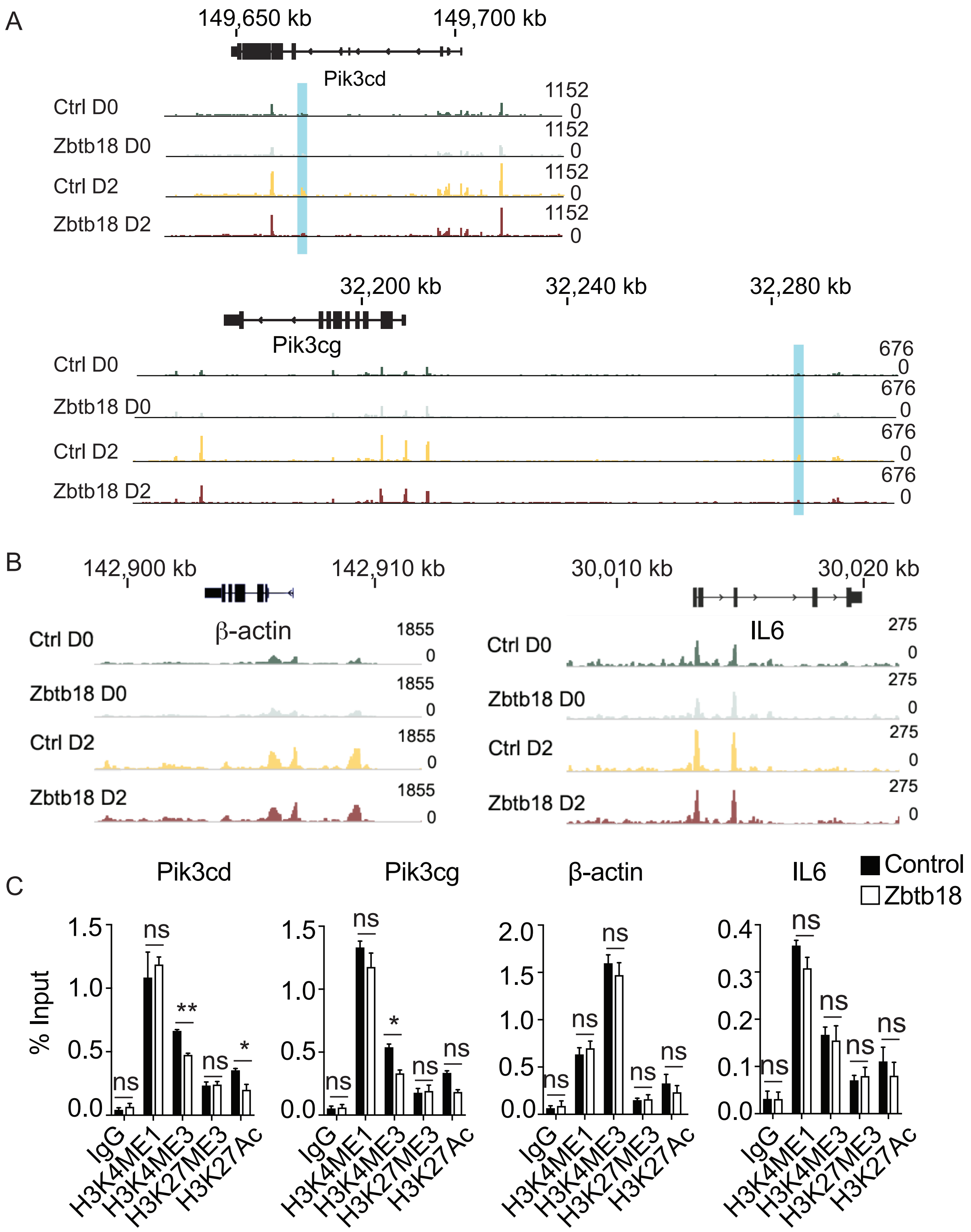

Supplementary figure 3. Constitutive expression of Zbtb18 affects histone modification and transcription of PI3K class I genes. (**A, B**) Normalized ATAC-seq coverage tracks (FPKM) for representative genomic regions at Pik3cd and Pik3cg locus (**A**) and b-actin and IL6, used as controls (**B**). Differentially accessible peaks are highlighted by blue shading. Read density tracks are from pooled replicates (n=2 for day 0, n=3 for day 2). (**C**) Chip-qPCR analysis of the ATAC differential peaks of Pik3cd and Pik3cg genes in Control or Zbtb18 transduced B cells two days after anti-CD40 and IL-4 stimulation (treated as described in Fig. 4A) by control IgG, H3K4me1, H3K4Me3, H3K27me3 and H3K27Ac antibodies. The promoter regions of β-actin and IL6 were used as negative controls. Data in **C** is from 3 independent experiments pooled together (n=3, biological replicates).

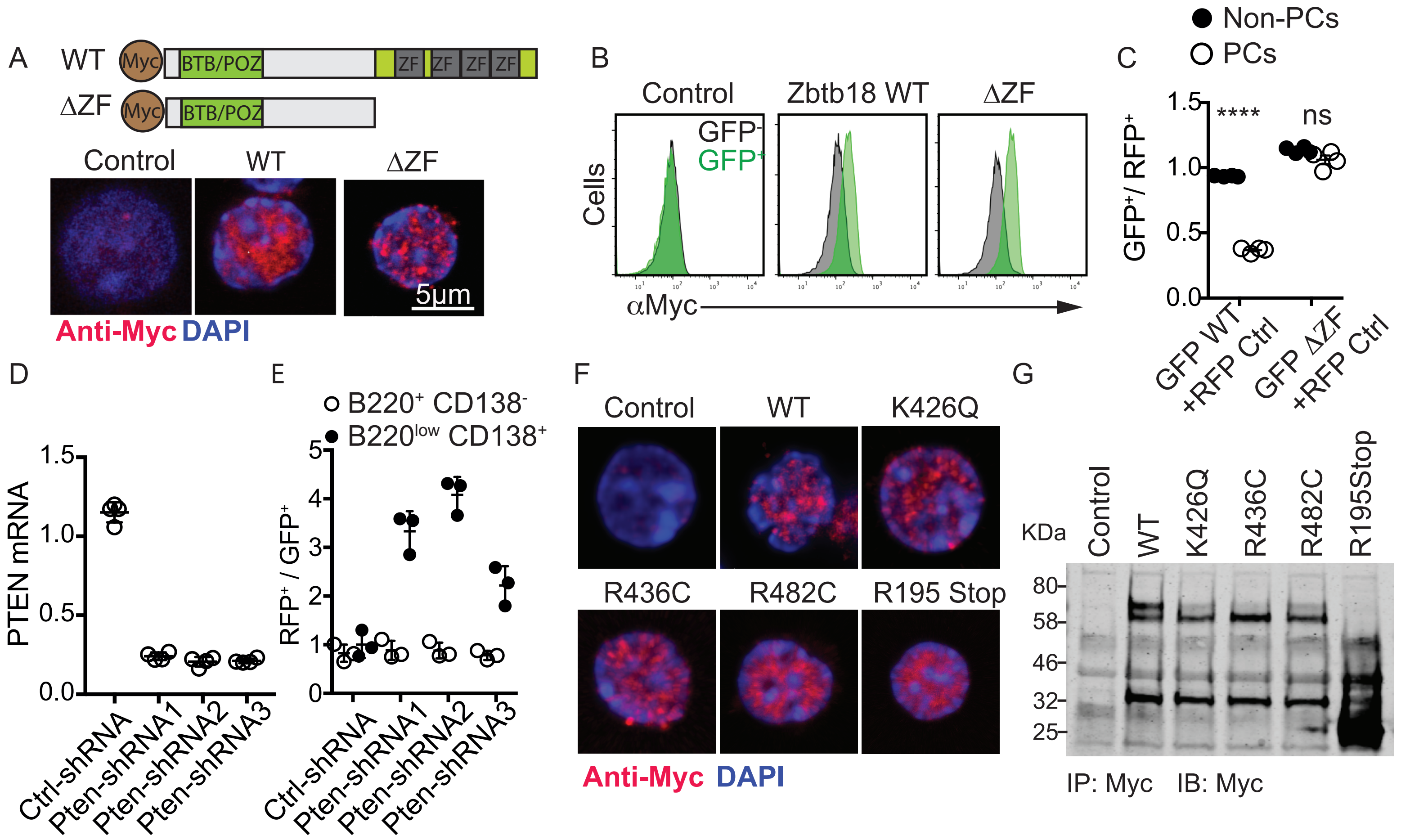

Supplementary figure 4. The zinc finger motif of Zbtb18 is important for its suppressive effect on PC differentiation. (**A**) Upper panel, schematic illustration of retroviral constructs of full length (WT) and truncated Zbtb18 (ZF) tagged with Myc. Bottom, Immunofluorescent analysis of B cells transduced with the above constructs. Transduced splenocytes were sorted ($B220^+ GFP^+$ cells) prior to analysis. (**B**) Splenocytes transduced with Control (GFP reporter alone), Zbtb18-Myc WT or ZF were intracellularly stained with anti-Myc. Histograms as pre-gated on the GFP^- (black) and GFP^+ (green) fractions, as indicated. (**C**) WT Zbtb18-Myc/GFP (WT GFP) or ZF Zbtb18-Myc/GFP (ZF GFP) transduced cells were co-incubated with control RFP transduced (RFP Ctrl) sorted B cells ($B220^+$ GFP^+ or RFP^+), stimulated with anti-CD40 and IL-4, and analysed 3 days later. Shown are the ratios between GFP and RFP expressing cells in the PCs ($B220^{low} CD138^{high}$) and non-PC ($B220^{high} CD138^{neg}$) subsets. (**D**) Quantitative PCR analysis of the transcript abundance of PTEN in GFP^+ sorted B cells ($B220^+$ GFP^+) transduced with either control-shRNA or different PTEN-shRNAs, relative to the abundance of GAPDH. (**E**) Frequencies of PCs ($B220^{low}$ $CD138^{high}$) and non-PCs ($B220^{high} CD138^{neg}$) in cells transduced with the indicated shRNA expressing vectors, 3 days post stimulation with IL-4 and anti-CD40. Data in **A-E** show one representative experiment out of 3 independent experiments performed with 3 or more technical replicates in each. In C, D and E, each circle represents one technical replicate. (**F**) Immunofluorescent analysis of B cells transduced with the indicated WT or mutated Zbtb18-Myc/GFP constructs. Transduced splenocytes were sorted ($B220^+ GFP^+$ live cells) prior to analysis. Control represents cells transduced with a GFP expressing reporter alone. (**G**) Immunoblot analysis of B cells transduced with the indicated Myc-tagged, or control constructs. Cells were sorted prior to analysis (gated on live B220+ GFP+ cells). Data in **F** and **G** from 3 independent experiments performed (n=1).
